# Species Richness and Distribution of Calliphoridae Along an Elevation Gradient in Sicily (Italy) and Ecuador

**DOI:** 10.3390/insects16050498

**Published:** 2025-05-06

**Authors:** M. Denise Gemmellaro, Gail S. Anderson, George C. Hamilton, Mariela Domínguez-Trujillo, Lauren M. Weidner

**Affiliations:** 1 Kean University, 1000 Morris Ave, Union, NJ 07083, USA; 2 Simon Fraser University, 8888 University Dr W, Burnaby, BC V5A 1S6, Canada; gail_anderson@sfu.ca; 3 Rutgers, The State University of New Jersey, 96 Lipman Drive, New Brunswick, NJ 08901, USA; hamilton@njaes.rutgers.edu; 4 Laboratorio de Entomología, Museo de Zoología QCAZ, Pontificia Universidad Católica del Ecuador, Quito 170143, Ecuador; mariela.adt@gmail.com; 5 Laboratorio de Zoología Terrestre, Instituto de Biodiversidad Tropical IBIOTROP, Colegio de Ciencias Biológicas y Ambientales, Universidad San Francisco de Quito USFQ, Quito 170901, Ecuador; 6 Arizona State University, West Campus, 4701 W Thunderbird Road, Glendale, AZ 85306, USA

**Keywords:** Calliphoridae, biodiversity, altitude, elevation, entomology, Diptera, forensic

## Abstract

Blow flies (Diptera: Calliphoridae) are among the first insects to arrive on a corpse, and so they are particularly important in forensic entomology. To use blow flies in forensic investigations, there must be information available on their species diversity, abundance, and distribution in the areas where investigations are conducted. Several factors can contribute to species distribution, and elevation is one of those factors. The purpose of this study was to document the distribution of Calliphoridae across four elevational gradients in Sicily and Ecuador, where little information is available. Baited traps were placed at elevations ranging from 20 m to 1552 m in Sicily (a major island and region of Italy and a distinct ecoregion) and 561 m to 3336 m in Ecuador. Species richness, relative abundance, and diversity were calculated, as well as the ratio of female to male blow flies and community assemblage. Twelve species were collected in Sicily, and seventeen species were collected in Ecuador. In Sicily, the most abundant species was *Lucilia sericata* (Meigen) (68.50% of the total capture), while in Ecuador, it was *Compsomyiops verena* (Walker) (51.67% of the total capture). In Sicily, significant differences were only observed in the relative abundance of *L. sericata* across elevations. In Ecuador, significant differences were observed in the relative abundance of *Calliphora nigribasis* (Macquart), *Chrysomya albiceps* (Wiedemann), *C. verena, Hemilucilia semidiaphana* (Rondani)*, Lucilia ibis* (Shannon), *L. purpurascens* (Walker), and *Paralucilia* sp. across elevations. These data can help build a checklist of blow fly species in these two regions and can be instrumental in environmental and forensic investigations.

## Simple Summary

This research describes two surveys conducted at different elevations in Sicily and Ecuador to assess the presence and distribution of blow fly species; larvae of these flies are important in forensic investigations because they feed on decomposing corpses. Baited traps were deployed at four different elevations in both regions to attract adult blow flies in order to analyze their species richness and relative abundance. In Sicily, a total of 12 different species were observed, with *Lucilia sericata* being the most abundant (68.5%); in Ecuador, 17 species were observed, with *Compsomyiops verena* being the most abundant (51.7%). Significant differences in relative abundance were observed across elevations for some species. The results of this research are valuable for medico-legal forensic entomology; they can help with the assembly of species checklists for these regions, which can then assist in the identification and analysis of blow fly species recovered from a body at death scenes. Moreover, a better understanding of the impact of elevation on species distribution can support environmental investigations as well as the assessment of changes in species ranges.

## 1. Introduction

Blow flies (Diptera: Calliphoridae) are the main initial arthropod decomposers of carrion [1]. Because of their necrophilous behavior, they are of great use to medico-legal entomology and forensic investigations and have therefore been the object of numerous studies in the literature.

Attracted by the Volatile Organic Compounds (VOCs) released by a decomposing body, adult blow flies are able to detect an exposed corpse shortly after death occurs [2,3]. In Colorado, De Jong [4] observed that a dead greyhound carcass was visited by adults of *Phormia regina* (Johann Wilhelm Meigen, 1826) 40 s after exposure and that oviposition occurred 13 min after exposure. Blow flies can travel several kilometers to reach carrion [5,6] and use it for their trophic needs as well as an oviposition substrate. Blow fly species, like *Lucilia cuprina* (Christian Rudolph Wilhelm Wiedemann, 1830), require a protein meal for the maturation of their ovaries [7]; a decaying corpse will provide such a meal and will also represent an efficient site for laying their eggs [8,9]. In South Africa, adults of *Chrysomya albiceps* (Christian Rudolph Wilhelm Wiedemann, 1819) and *Chrysomya marginalis* (Christian Rudolph Wilhelm Wiedemann, 1830) were observed traveling several kilometers to find a suitably sized carcass to lay their eggs [10]. For these reasons, blow flies are usually the most numerous insect group observed on decomposing remains. A study of arthropod succession on carrion in Venezuela reported that seven of the fourteen collected species of primary forensic importance were calliphorid flies [11]. A similar study in Vienna, Austria, traced insect presence on a decomposing carcass for 60 days and observed that blow flies were the most abundant in the initial 15 days [1].

The functional roles of blow flies in ecosystems are diverse. Besides recycling carrion, several species use feces as a protein source and can therefore be important vectors of enteric pathogens [12,13]. They can also colonize living hosts (humans or other vertebrates); this phenomenon is known as myiasis and can have a strong impact on the livestock industry [14]. Blow flies are important pollinators; studies have analyzed their role in the pollination of different crops, including carrots [15] and Brussels sprouts [16], both wild-growing and in cages [17,18], hypothesizing a potential use of calliphorids as managed pollinators.

The most well-known role of Calliphoridae remains the one they play in forensic entomology. Since they are the first individuals to arrive and colonize a body shortly after death [19,20], blow flies are also among the main necrophagous insects to be used in forensic investigations and can be used to estimate the minimum post mortem interval (mPMI), defined as the time elapsed from the colonization of the body to its discovery [21,22,23]. In order to use blow flies appropriately in forensic entomology, it is essential to have information on their species diversity, abundance, and distribution for each geographic area where investigations are conducted. Studies have analyzed information about blow fly species composition and community structure in different areas around the world, including North America [24,25,26], Europe [27,28,29,30,31], and Asia [32]. Although some of this work analyzed the spatial and temporal distribution of blow flies [26,29,31], very little work has been done focusing on the elevational distribution of such flies [33,34,35,36]. Hodecek and Jakubec [37] examined 160 criminal investigations from 1993 to 2007 in Switzerland, where entomological evidence was used in the cases. They examined the effect of elevation on the presence or absence of species, eliminating the effect of seasons. They discovered that elevation is significantly associated with the occurrences of blow flies; for example, increases in the number of *Calliphora vomitoria* (Carl Linnaeus, 1758) were highly correlated with increasing elevation. *Calliphora vomitoria* and *Lucilia caesar* (Johann Wilhelm Meigen, 1858) showed a significant change in presence with elevation (higher elevations were preferred by *C. vomitoria,* while lower elevations were preferred by *L. caesar*); however, elevation seemed to have an effect on other species as well.

Species distribution across different elevations has been examined for several taxa and has shown that there are two commonly observed relationship patterns between elevation and species richness. The first pattern is known as a monotonic decrease, where species richness decreases as elevation increases [38,39]. The second most common pattern is a hump-shaped pattern, where species richness peaks at intermediate elevations [40,41,42,43,44,45,46,47,48]. These patterns have been shown to exist across various forms of taxa, including fungi [49], vascular plants [50], birds [51], mammals [52], and insects [39,41,44,53,54,55,56,57,58,59]. The distribution of species across elevational gradients can be impacted by several biotic and abiotic factors (e.g., temperature, humidity, and human and non-human animal activity, including deforestation and urbanization). Numerous abiotic factors play a role in the relationship between species distribution and elevation; among them are physical geometric constraints [60,61] and climate [62,63]. If species are constrained by hard boundaries (large bodies of water), the bottom of a valley (lower boundaries), or eco-physiological features such as elevation increase (upper boundaries) [64], they may face an actual barrier to dispersal [61].

On a global level, anthropogenic climate change over the period from 1850–1900 to 2010–2019 has resulted in a temperature increase of 0.8 °C to 1.3 °C, with a best estimate of 1.07 °C. The predicted climate response to greenhouse gas emissions results in a best estimate of warming for 2081–2100 that spans a range from 1.4 °C to 4.4 °C [65]. By 2100, an increase in temperature of 1.4–5.8 °C has been estimated, compared to 1990 [66]. Along with warmer temperatures, seasonal changes in precipitation, radiation, potential evapotranspiration, and other climatic regimes are predicted [67]. Studies performed across different taxa have shown that the duration of life cycle stages was affected in response to climatic changes throughout the year [68,69,70] and that their distributions shifted towards higher latitudes or higher elevations [71,72,73,74]. Furthermore, it was shown that rising temperatures may bring new ecological interactions (e.g., new species introduction, new predator–prey interactions, or new plants that do not provide enough nutrients) [75], as well as alterations of existing interactions due to differences in thermal sensitivities [76]. These potential climatic changes can dramatically impact species distribution, as species are expected to respond to these changes at the species level [77]; moreover, the specific thermal biology of blow fly species may help in understanding their distribution in both micro-environments and on a larger, global scale [78]. The effects of climatic impact on insects of forensic importance have recently been investigated, and it was asked the biodiversity of forensically relevant taxa be surveyed and monitored to establish a baseline for their occurrences [79,80].

Better knowledge of the distribution of blow fly species across elevational gradients in areas rich in biodiversity will improve our understanding of the biology and behavior of this group. This information will provide not only a tool for ecological investigations of climatic changes, but also for forensic investigations. Information on biodiversity and distribution of these taxa will be valuable in medico-legal entomology, particularly when using insects to estimate the minimum post-mortem interval or the time of colonization. Knowing the probable species composition at a specific location and season can indeed improve the forensic significance of those estimations; moreover, the occurrence of indicator species can be an essential step towards better understanding of how the environment influences insect colonization. Therefore, the purpose of this study was to document the distribution of forensically important Calliphoridae across elevational gradients in two different areas, Sicily and Ecuador, for which little information is available.

## 2. Materials and Methods

### 2.1. Sampling Procedure

Sampling was performed at all sites using different aerial traps baited with beef liver (Sicily) and a mixture of chicken products (Ecuador); while both types of bait are reported in the literature [81], bait was chosen based on availability in each location. Traps were baited on site, hung from trees at an average height of 1.8 m ± 0.12 m (SE), and left at the site for one week. During the sampling periods, any traps that were lost or stolen were not replaced. Two one-week sampling sessions were conducted in July of 2017 in Sicily and in July of 2016 in Ecuador; the different sampling times were due to practical reasons. At the end of each sampling period, the traps were collected and emptied. Adult Diptera were cleaned with water, left to dry, placed in vials, and frozen until they were sorted by family and pinned. Non-dipteran arthropods were not considered in this survey. Identifications were performed using morphological characteristics with several keys [82,83,84,85]. Sex was determined based on the distance between their eyes; the space between the eyes of a female is broader than what is observed in males [86]. Datasets for the two locations can be viewed at https://doi.org/10.5281/zenodo.7062111 and https://doi.org/10.5281/zenodo.7060325.

### 2.2. Study Areas

**Figure 1:**
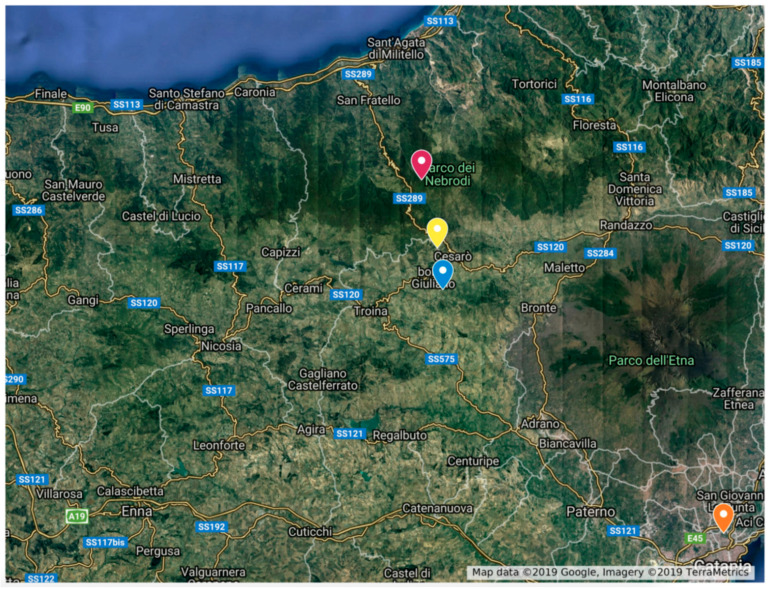
Sampling sites at different elevations in Sicily. Orange = 20 m (level 1), blue = 700 m (level 2), yellow = 1153 m (level 3), and red = 1552 m (level 4).

**Figure 2:**
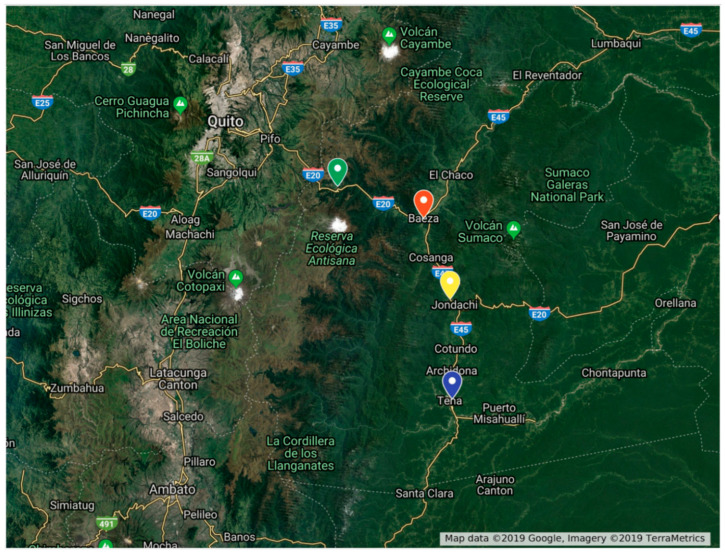
Sampling sites at different elevations in Ecuador. Blue = 561 m (level 1), yellow = 1312 m (level 2), red = 1948 m (level 3), and green = 3336 m (level 4).

The survey was conducted in the eastern part of Sicily, on the slopes of Mt. Etna (Figure 1), and in the inner part of Ecuador (Figure 2). These areas were chosen because both locations were accessible, had substantial changes in elevation, and lacked information on the biodiversity and distribution of forensically important blow flies. All sites are also listed in Table 1.

Level 1 (37.5427° N, 15.0843° E, Elevation 20 m): This site is located in a green area populated by moderate Mediterranean vegetation (macchie or garrigue), *Cistus* sp. (Cistaceae), and *Senecio* sp. (Senecioneae) near the city of Catania, which has some of the highest seismic activity in the country [87]. It is located on the southeastern slope of Mt. Etna and is one of the more populated cities in Sicily, with 314,000 people in 2017 (ISTAT, National Institute of Statistics).

Level 2 (37.8071° N, 14.6994° E, Elevation 700 m): This site is located in the area of San Teodoro, a small village in the Province of Messina, on the western side of Mt. Etna. The area is covered by Mediterranean scrubs, almond trees (*Prunus dulcis*, Rosaceae), and willows (*Salix* sp. Salicaceae).

Level 3 (37.8521° N, 14.6919° E, Elevation 1153 m): This area is known as *Condrada Casale Nuovo*, though it is still part of the San Teodoro municipality. This rural area is covered in Turkey oaks (*Quercus laevis* (Thomas Walter) Fagaceae) and elders (*Sambucus nigra* (Carl Linnaeus) Adoxaceae).

Level 4 (37.9258° N, 14.6702° E, Elevation 1552 m): This rural site is on *Monte Soro*, the highest peak of the Nebrodi Mountains, located northwest of Mt. Etna. The most abundant trees in this area are beech trees (*Fagus* sp.).

Level 1 (0.5956° S, 77.5011° O, Elevation 561 m): This site is part of a megathermal rainforest and is located in the area around Tena, in the valley of the Misahuallí River, in the Amazon region. Tree crowns, in this area, are not commonly stratified because they belong to a large number of species, whose maturation period and reaction to light is different.

Level 2 (0.4143° S, 77.4837° O, Elevation 1312 m): This site is located in Sarayacu (1312 m), situated in a tropical rainforest in the Amazon region. In this area, trees are typically quite diverse, and gregarious dominants are usually not common. Tropical rainforest is often defined as multilayered, since the tree crowns form distinct strata.

Level 3 (0.282.31° S, 77.533365° O, Elevation 1948 m): This site is located in the area around Baeza in the central northern part of the Ecuadorian Amazon Forest. The climate is equatorial, semi-humid, and mesothermic. This area is incredibly diverse, and in a one-hectare plot of land, it is possible to find 40 to 100 species of trees [88].

Level 4 (0.222324° S, 78.82235° O, Elevation 3336 m): The highest of the Ecuador sites, this location is in Papallacta, a mountainous area of the eastern Cordillera whose ecosystems range from tropical forests to alpine/glaciers as the elevation increases. It is characterized by an equatorial, high-mountain climate, and one of the most well-known species of this area is the paper tree (*Polylepis* sp.), whose height can reach up to 10 m.

## 3. Data Analysis

Calliphorid species richness (number of species), relative abundance, and community structure (occurrence and abundance) were analyzed for each region and elevation. All data were subjected to a Shapiro–Wilkes test to determine if the raw data were normally distributed prior to analysis. This test revealed that relative abundance in both Sicily and Ecuador was not normally distributed; richness was not normally distributed in Ecuador but had a normal distribution in Sicily. The relationship between species richness/abundance and elevation was analyzed using a Kruskal–Wallis test. This nonparametric test is an alternative to a one-way (between groups) ANOVA and is commonly used to compare three or more sample ranks instead of means. The null hypothesis tested was that samples used for comparisons were drawn from the same distribution or from distributions having the same median [89]. To analyze species richness in Sicily, which was normally distributed, an ANOVA was performed. When significant results were obtained, a post hoc test was performed; significant results after the Kruskal–Wallis analysis were further analyzed with a Dunn test [90] with the Benjamini–Hochberg adjustment for pairwise comparison [91], while significant results obtained after the ANOVA were further analyzed through a Tukey’s HSD test. For each elevation level within each ecoregion, the Shannon (*H*) [92] diversity index and the Sørensen’s Similarity Index CC [93] were calculated. Sørensen’s Similarity Index CC is an index that compares the species composition of two sites, taking into account the species present in each of the two sites and the species each have in common, and ranges between 0 and 1, with 0 being completely dissimilar and 1 being completely similar.

Considering the differences in sample size across elevations, we also performed a rarefaction/extrapolation analysis using the iNEXT online software developed by Dr. Anne Chao [94,95]. This software computes and plots rarefaction curves, allowing users to compare diversity estimates across different samples by standardizing data to a common sample size or coverage. Rarefaction curves allow the visualization of the expected number of species (richness) for a given sample size [96,97]. For the analysis, we selected the following parameters: Abundance data type, Diversity order q = 0 (Species Diversity), Bootstraps = 50, Confidence interval = 0.95.

Using the methods developed by Dufrêne and Legendre [98] and used by Weidner et al. [26], a nonmetric multidimensional scaling (NMDS) analysis followed by a multi-response permutation procedure (MRPP) for multiple pairwise comparison [99] and an indicator species analysis (ISA) were performed. A multi-response permutation procedure (MRPP) is a permutation test that assesses differences in two or more groups by comparing the distance between the sample units in each group. It is commonly used when assessing whether the distributions of such groups are similar or different. The null hypothesis is that there is no difference between the groups. An indicator value is used to indicate which taxon or taxa are considered the best predictor of that elevation; a value of 0 shows that they are a poor predictor, and a value of 100 shows a perfect predictor. In order to sort species and examine their degree of association, a cluster analysis based on abundance was performed. Distances between clusters are determined by the greatest distance between any two items in different clusters. Analyses were conducted using R Studio 1.1.463 [100], Microsoft^®^ Excel 2018 version 16.21.1, and Google Colab [101].

## 4. Results

Average temperatures (±SE), average humidity (±SE), and total precipitation in each location were recorded (Table 2).

### 4.1. SICILY

A total of 9923 adult Diptera were collected; of these, a total of 4926 belonged to the family Calliphoridae, encompassing 12 species within four genera (Table 3). The most abundant calliphorid species overall was *Lucilia sericata* (68.50%). The least abundant species was *Cynomya mortuorum* (Carl Linnaeus, 1761) (0.02%).

The elevation where the highest number of blow flies were collected was 700 m (3108 individuals), followed by 1153 m (1150 individuals), 1552 m (397 individuals), and 20 m (206 individuals). The predominant species at 20 m were *L. sericata* and *Calliphora vicina* (Jean-Baptiste Robineau-Desvoidy, 1830). At 700 m, the dominant species was *L. sericata*. At 1153 m, *C. vicina,* followed by *L. sericata,* were the two most abundant species. At the highest elevation (1552 m), *Calliphora vomitoria* and *C. vicina* were the most collected species (Table 3). The sex ratio of calliphorids collected showed an overall bias towards females, with an overall m/f ratio of 0.15. The m/f ratio for the two most common species was 0.05 and 0.54 for *L. sericata* and *C. vicina*, respectively (Table 4).

**Figure 3:**
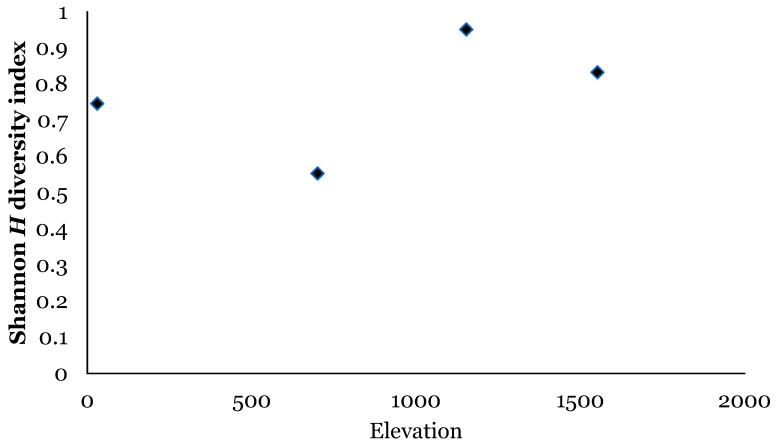
Blow fly species diversity across elevations in Sicily using the Shannon (*H*) diversity index.

**Figure 4:**
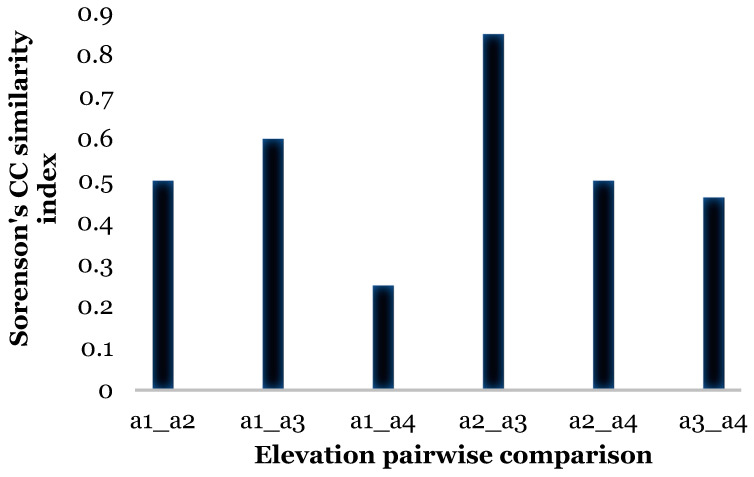
Sorenson’s (CC) Similarity Index between elevations in Sicily. a1 = level 1 (20 m), a2 = level 2 (700 m), a3 = level 3 (1153 m), and a4 = level 4 (1552 m).

**Figure 5:**
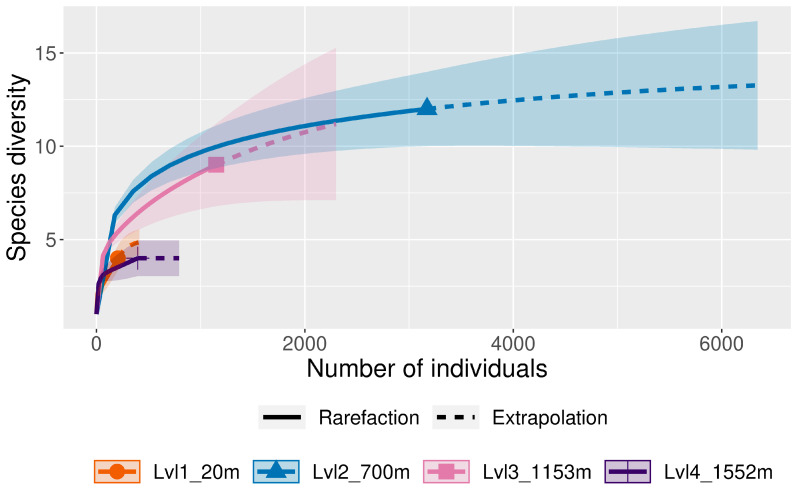
Rarefaction/Extrapolation curves for the four elevation levels (lvl) in Sicily; the x-axis displays the numbers of specimens and the y-axis the species richness (number of species).

**Figure 6:**
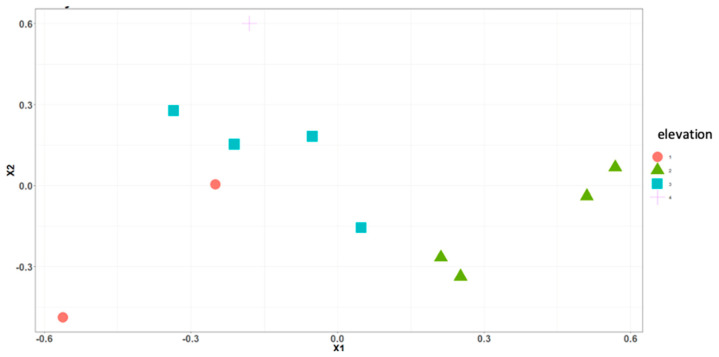
Nonmetric multidimensional scaling ordinations of blow fly communities across elevations in Sicily. This ordination explains 91% of variation (minimum stress = 0.083). MRPP analysis showed significant differences between 700 m and 1153 m (A = 0.3035 *p* = 0.035). Circles represent elevation 20 m, triangles elevation 700 m, squares elevation 1153 m, and crosses elevation 1552 m.

There were significant differences in relative abundance across elevations for only one species, *L. sericata*. Specifically, significant differences in *L. sericata* relative abundance were found when comparing 20 m with 700 m and 700 m with 1550 m (Table 5). The Shannon diversity index (H) was calculated for all four elevation levels (Figure 3 and Figure 11). Sørenson Similarity Index (CC) for each pair of elevations is represented in Figure 4. Since species richness was normally distributed across elevations (Shapiro–Wilkes test, *p* = 0.313) an ANOVA was performed. The test indicated significant differences between elevations, specifically between 20 m and 700 m, 20 m and 1153 m, and 700 m and 1552 m. Rarefaction/extrapolation curves were also generated to assess species diversity in relation to sampling effort across the four elevations (Figure 5). Level 1 exhibited a steep initial increase in diversity with a solid curve and minimal extrapolation, indicating high diversity accumulation at low sampling effort and suggesting that additional sampling may still uncover new species, as the curve had not yet plateaued. Level 2 showed a similarly steep initial rise, followed by a partial plateau during rarefaction and a slight upward trend in the extrapolated segment, implying moderate sampling completeness with potential for discovering additional taxa. Level 3 revealed a continuously increasing curve, steeper than Level 1, with extensive rarefaction and extrapolation but no clear plateau, reflecting a community with high diversity that remains undersampled. In contrast, Level 4 displayed a short but steep curve that reached a plateau after extrapolation, suggesting a smaller but more fully sampled community. Together, these patterns highlight variability in species richness and sampling completeness across the datasets. Coverage-based and size-based rarefaction/extrapolation data as well as the sample completeness curve and the coverage-based rarefaction and extrapolation sampling curves are available in the supplementary materials. *Lucilia sericata* and *Lucilia silvarum* (Johann Wilhelm Meigen, 1826) were indicator species for 700 m (Level 2), while *C. vicina* was an indicator species for 1153 m (Level 3); no indicator species were found for 20 m (Level 1) or 1552 m (level 4) (Table 6). Blow fly communities were shown to be significantly different between elevations, with significant differences between 700 m and 1153 m (MRPP: A = 0.304, *p* = 0.035) (Table 7, Figure 6). “A” is known as the “effect size” or the “chance-corrected within-group agreement”; A = 1.0 can be read as perfect agreement between the groups, and A = 0 can be read as “no better than expected by chance” ([102], p. 1124); lower values indicate differences between the groups.

### 4.2. Ecuador

A total of 2890 adult Diptera were collected; of these, 1492 calliphorids belonged to nine genera and seventeen species. The most abundant calliphorid species collected during the survey was *Compsomyiops verena* (Francis Walker, 1849). The least abundant species collected were *Cochliomyia hominivorax* (Jean Charles Coquerel, 1858), *Chrysomya* sp. and *Lucilia ochricornis* (Christian Rudolph Wilhelm Wiedemann, 1830) (constituting 0.06%, corresponding to one individual captured per species). The largest number of flies was collected at 3336 m (550 flies) and 1948 m (550 flies). In the remaining sites, the flies collected were 215 at 561 m and 130 at 1312 m (Table 8).

The predominant species at 561 m were *Paralucilia* sp., *Lucilia eximia* (Christian Rudolph Wilhelm Wiedemann, 1819), and *C. albiceps*. At 1312 m, predominant species were *C. albiceps*, *L. eximia,* and *Paralucilia* sp. At 1948 m, predominant species were *C. verena*, followed by *Lucilia purpurescens* (Francis Walker, 1836). At the highest elevation (3336 m), the two main species were *C. verena* and *Calliphora nigribasis* (Pierre-Justin-Marie Macquart, 1851) (Table 8). The sex ratio (m/f) of the blow flies collected has an overall value of 0.25. The m/f ratio of the four most abundant species was 0.27 for *C. verena*, 0.66 for *C. nigribasis*, 0.12 for *L. eximia,* and 0.24 for *C. albiceps* (Table 9).

Significant differences were observed across elevations for *C. nigribasis*, *C. albiceps*, *C. verena*, *H. semidiaphana*, *Lucilia ibis* (Raymond Corbett Shannon, 1926), *L. purpurascens*, and *Paralucilia* sp. *Calliphora nigribasis* numbers differed significantly between level 1 and level 4, and level 2 and level 4. The distribution of *C. albiceps* was significantly different between level 1 and level 3 as well as level 1 and level 4. *Compsomyiops verena* was significantly different between level 4 and level 1. The distribution of *L. ibis* was significantly different between level 1 and level 3. *Lucilia purpurescens* was significantly different between level 1 and level 3, level 2 and level 3, and level 3 and level 4. For *Paralucilia* sp., significant differences were observed between level 1 and level 3, and level 1 and level 4 (Table 10).

**Figure 7:**
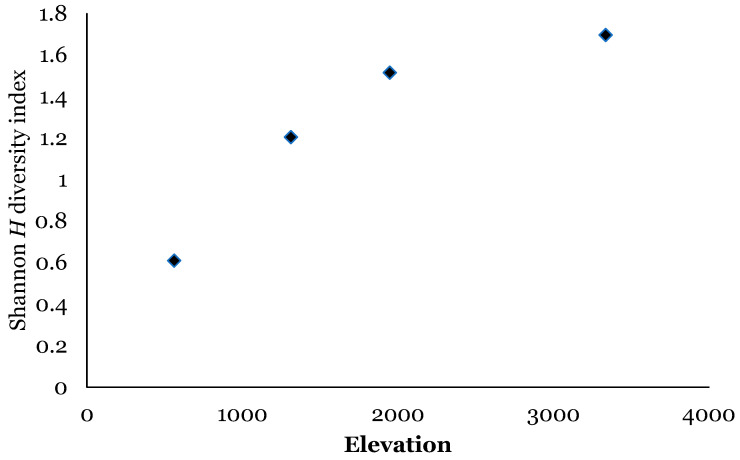
Blow fly species diversity across elevations in Ecuador using the Shannon (*H*) diversity index.

**Figure 8:**
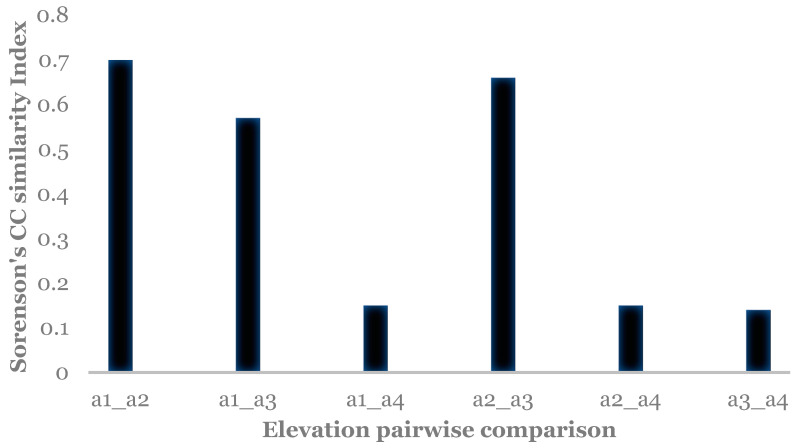
Sorenson’s (CC) Similarity Index between elevations in Ecuador. a1 = level 1 (561 m), a2 = level 2 (1312 m), a3 = level 3 (1948 m), and a4 = level 4 (3336 m).

**Figure 9:**
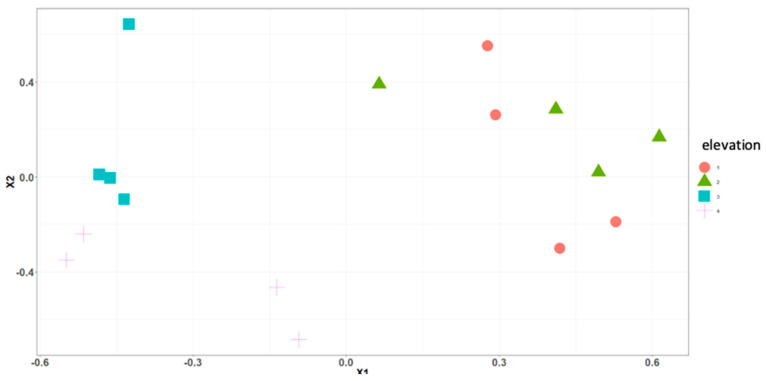
Nonmetric multidimensional scaling ordinations of blow fly communities across elevations in Ecuador. This ordination explains 82% of variation (minimum stress = 0.14). MRPP analysis showed significant differences between all levels (561 m and 1948 m (A = 0.2893 *p* = 0.027); 561 m and 3336 m (A = 0.2747 *p* = 0.04); 1312 m and 1948 m (A = 0.31261 *p* = 0.025); 1312 m and 3336 m (A = 0.2977 *p* = 0.028)), except between 1948 m and 3336 m (A = 0.1031 *p* = 0.083). Circles represent elevation 561 m, triangles elevation 1312 m, squares elevation 1948 m, crosses elevation 3336 m.

**Figure 10:**
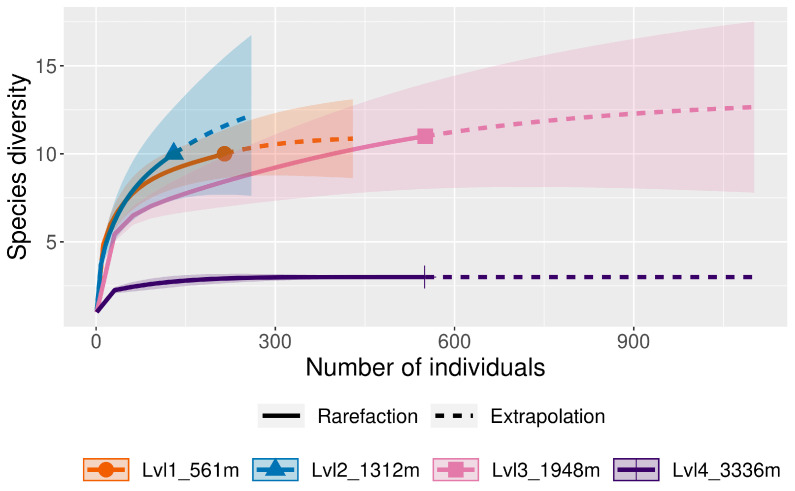
Rarefaction/Extrapolation curves for the four elevation levels (lvl) in Ecuador; the x-axis displays the numbers of specimens and the y-axis the species richness (number of species).

**Figure 11:**
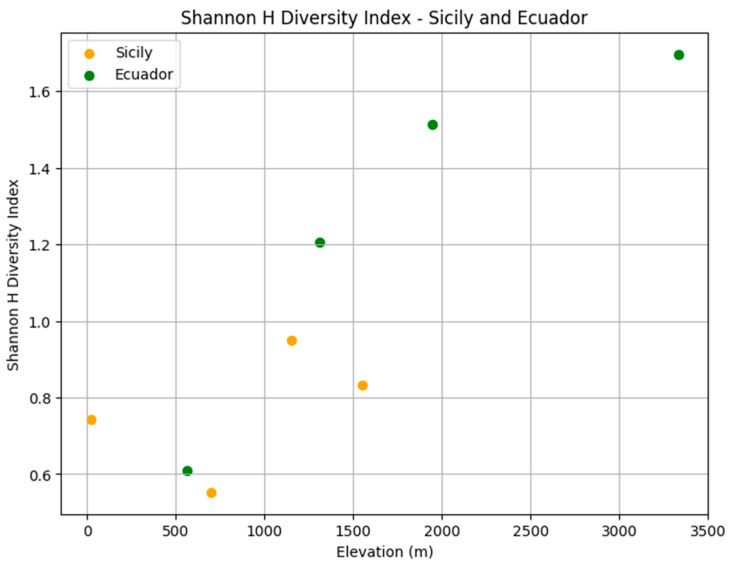
Blowfly species diversity across elevations in Sicily (orange) and Ecuador (green) using the Shannon (*H*) diversity index.

The Shannon diversity index (*H*) was calculated for all four elevation levels (Figure 7 and Figure 11). The Sorenson Similarity Index (CC) for each pair of elevations is represented in Figure 8. Species richness was not normally distributed across elevations (Shapiro test *p* = 0.034); therefore, a Kruskal–Wallis analysis was performed, which showed no significant difference across all elevations. The Indicator Species Analysis (ISA) showed that *Paralucilia* sp. was found to be a strong indicator of the first elevation level, *L. purpurascens* and *L. ibis* were strong indicators for the third elevation level, and *C. nigribasis* was a strong indicator for the highest elevation (Table 11). Blow fly communities were shown to be significantly different across elevational gradients, with significant differences between all levels except between levels 1 and 2, and levels 3 and 4 (Table 12, Figure 9). Rarefaction curves were generated to assess species diversity in relation to sampling effort across four elevations (Figure 10). The x-axis represents the number of individuals sampled, while the y-axis reflects species richness (number of species). Level 1 shows a steep initial increase in diversity, followed by slight tapering, suggesting relatively high richness with some unsampled diversity remaining. Level 2 showed a more gradual slope and began to plateau moderately, indicating a moderate level of species richness with a nearing saturation point. Level 3 displayed the least steep curve, plateauing quickly, which suggests relatively low species richness that was sufficiently captured with the available sampling effort. Level 4 showed a somewhat steep ascent with minor leveling off toward the end, indicating moderate richness and an intermediate level of sampling completeness. Overall, the differences in the shape and extent of rarefaction among datasets suggest variability in community complexity and sampling completeness. Coverage-based and size-based rarefaction/extrapolation data as well as the sample completeness curve and the coverage-based rarefaction and extrapolation sampling curves are available in the supplementary materials.

## 5. Discussion

Our study shows a correlation between elevation and blow fly distribution, differences in species richness across elevation levels that can be related to vegetation and/or urbanization levels and has produced a reference baseline for Calliphoridae communities in both Sicily and Ecuador.

With few exceptions, the elevational distribution of blow flies in the Mediterranean ecoregion has not been extensively researched. Baz et al. [35] analyzed the elevational distribution patterns of blow flies in Spain at nine different elevations and found eight different species with significant differences between elevation and abundance for most of the species collected. In Sicily, Gemmellaro et al. [103] looked at blow fly colonization inside volcanic caves and surrounding areas during periods of cold temperatures and noticed a delay in hypogenic colonization by *C. vicina* and *C. vomitoria*. This study shows that blow fly species distribution can be correlated with changes in elevation. In fact, the data collected during our survey show that the relative abundance of *L. sericata*, the most abundant species collected in Sicily (68.5% of total capture), changed significantly throughout the elevation gradient, going from being 42.23% of capture at 20 m to being absent at the highest elevation (1552 m). These differences could be due to the fact that, along with elevation, average temperature and habitats changed across sites, going from an urbanized location with a mean temperature of 25 °C to a more rural area with abundant vegetation and a mean temperature of 16 °C. This is in agreement with previous studies that have found *L. sericata* mainly in urban sites [26]. However, this survey documented this species also at intermediate elevations in rural sites; this corresponds with other studies, which have reported *L. sericata* in less urbanized rural areas [104].

*Calliphora vicina* was the second most abundant species collected in Sicily (21.0% of total capture), and its relative abundance, although not significantly different across elevations, still showed variation in the survey, with higher values observed at low and high elevations (56.8% at 20 m, 50.6% at 1153 m, and 40.8% at 1552 m). This pattern is consistent with data found in the literature, which classifies *C. vicina* as an urban species [105], although it has been observed in non-urban scenarios as well [106]. Moreover, the higher presence of *C. vicina* at an elevation where the temperature was lower agrees with the definition of *C. vicina* as a thermophobic species [107] and with studies on the geographical distribution of blow flies, which have shown that this species is more abundant in northern, colder regions [26].

At the highest elevational level (1552 m), *C. vomitoria* was the main colonizer, representing 55.16% of the total number of flies captured; at lower elevations, however, this species had low representation. *Calliphora vomitoria* is also considered a species well adapted to cold climates [108], and its higher abundance at the highest elevation, when compared to *C. vicina*, may be related to the fact that *C. vomitoria* prefers non-anthropic habitats, while *C. vicina* prefers urban environments [109]. The highest site of this survey was characterized by low urbanization and colder temperatures, providing a suitable habitat for this species. The comparison of male/female ratios of the two most abundant species showed large differences. Males represented 5% of the total capture for *L. sericata* and 35.5% for *C. vicina*. In another survey conducted in Manhattan (NY) with baited traps, high numbers of *C. vicina* males were also recorded [110]. This observation might be explained by the nutritional needs of *C. vicina* males, which, when offered multiple food sources, have been shown to prefer protein-rich substrates over carbohydrates [111].

While we know that this study only provides a compartmentalized view of the blow fly populations present in the areas, it is possible that the male/female ratio we observed may mirror the actual ratio of the local population. There are several theories that try to explain sex ratios in various groups; female-biased sex ratios have been observed in several social insects, where collaboration among females is common. According to Hamilton’s local mate competition (LMC) theory [112], a female-biased sex ratio in groups where mating occurs locally can be explained by the resulting reduction in competition among related males and the increase in the availability of females [113].

In Sicily, the most diverse sampling site was located at elevation level 3–1153 m (*H* = 0.95); this could be related to the habitats provided by the geographical features and vegetation of the site. The lowest diversity was observed at elevation level 2–700 m (*H* = 0.55); however, this was also the site where the highest number of blow flies was collected. The similarity CC index for the Sicily sites showed that species composition in relation to elevation is most dissimilar between 20 m and 1552 m (CC = 0.025); different urbanization and average temperatures characterize these areas, creating habitats that can be conducive to different blow fly species. The most similar elevations in relation to diversity were 1153 m and 1552 m (CC = 0.85), where similar landscapes and vegetation are present, and the anthropogenic impact is low.

Species richness was significantly different between the first elevation level (20 m) and the second (700 m), and between the second (700 m) and the third (1153 m) levels. This may be explained by the location of the first level being in close proximity to a large city, while none of the others were. Richness was also the same at the lowest and highest elevations (richness = 4), even though there was only one species in common between the two levels (*C. vicina*). The number of flies captured during the survey was also lower at the two extreme elevations (206 at 20 m and 397 at 1552 m) than it was at the two intermediate ones (3197 at 700 m and 1153 at 1153 m). This is in agreement with numerous authors who have found the correlation between species richness and elevation to have a hump-shaped pattern, with a peak in richness at intermediate elevations [61,114].

In Ecuador, the number of blow flies collected during the survey was lower than what was collected in Sicily. The same was also true when looking at all Diptera captured during the study (9923 for Sicily; 2890 for Ecuador). This could be related to the high relative humidity and precipitation in the sampling sites in Ecuador. High humidity can be inversely related to fly abundance [115]. Research in Malaysia showed that the abundance of Muscidae diminished as humidity increased [116]. However, the number of blow fly species was slightly higher in Ecuador (17 versus 12 species in Sicily). This could also be due to the fact that the former is an island, while the latter is located on the mainland. Similar results have been observed in other studies comparing insect diversity between islands and the mainland, where significant differences were found in both the number of species and in the number of individuals that were collected [41]. Moreover, when compared to Sicily, Ecuador is closer to the Equator, and studies have noticed a higher number of species at lower latitudes, and this has been correlated with warmer climates and to other factors (such as vegetation, human footprint, and physical barriers) [117]. The sites sampled in Ecuador had more diverse vegetation and lower levels of urbanization when compared to the Sicilian sites, which could explain the difference in the number of species sampled in the two locations.

In Ecuador, just over half of the total capture was represented by *C. verena* (51.7%). No extensive data are available in the literature about the biology and development of this blow fly. It is known to be attracted to garbage and decomposing matter, and occasionally feces [34]. In a survey of the blow fly community of Peru, Greenberg and Szyska [118] collected this fly at an elevation of 1430 m, in an asynanthropic area, and recorded an on-site egg-to-adult development of 17 days. In this survey, *C. verena* was also the most abundant blow fly collected at the highest site in our survey (Papallacta, 3336 m), which is also the site where the highest number of flies was collected. This is surprising considering that Papallacta was the most elevated site of this survey, but it could be explained by the low anthropogenic impact that characterizes this site and by the different habitats that the great diversity of this area provides.

Significant differences in abundance across all four elevational levels were observed for *C. nigribasis*, with few collections at the highest elevation (3336 m). *Calliphora nigribasis* has been observed to be a good flier and can travel up to 3.5 km a day [119]. The fact that it was only captured at the highest elevation in our survey could be interpreted as a preference for this habitat. It could also be that geographical constraints, such as mountains, are a physical impediment to the dispersal of this species. The distribution of *C. albiceps* and *Paralucilia* sp. was also significantly different and seemed to prefer lower elevations (561 m and 1948 m) given that they were not collected at the higher sites.

The Shannon diversity index indicated that the most diverse site was the highest site, Papallacta (3336 m) (*H* = 1.7). Overall, the habitats in this area were quite diverse, ranging from tropical forest to glaciers and, therefore, may provide living conditions for numerous species. Sorenson’s Similarity Index showed that the species composition at the highest elevation is quite dissimilar to all the other levels, and that the two lower elevations and the two intermediate elevations were very similar to each other. However, species richness was not significantly different across the four elevations, indicating that even though species may differ across elevations in relation to habitats and temperature, their numbers do not change significantly.

One of the limitations of this survey was the use of bait as opposed to whole animal carcasses. Bait type and size may have impacted the number and composition of the flies collected. This possibility has been suggested by other studies that compared the use of bait as opposed to a carcass [99,120,121]. Additionally, the rate of decay of the liver bait used for this survey may have been impacted by the different environmental parameters at each elevation. This difference in decomposition could have affected the chemical cues released and, therefore, affected the ‘attractiveness’ of the bait. A study conducted by Vogt and Woodburn [122] reported significant differences in the number of flies collected by seven-day-old bait compared to fresh bait. Another potential limitation may be due to the different types of bait used in the two ecoregions, as this difference may have impacted the flies that were collected; however, Farinha et al. [123] conducted a survey using three different baits and noticed that only one of the forensically important species they collected (*L. sericata*) was significantly more present in one of the baits (pork liver). Furthermore, in one study where different types of bait were used in the same location, the major taxa of forensic interest appeared to be attracted to all types of bait, although they did show a preference for a specific one [81]. Additionally, conducting two surveys in different years may have also affected the results we observed. Future work should consider analyzing multiple seasons across multiple years along elevational gradients to provide useful information about the seasonality of the species collected.

## 6. Conclusions

The results of these surveys provide baseline data about the calliphorid communities in Sicily and Ecuador across different elevations and can help in the interpretation of general ecological patterns of calliphorid species distribution across elevations. Moreover, this information could be used as a reference in future studies, as well as assist with increased implementation of the use of insects in forensic investigations. Knowing which species are commonly present in specific geographical areas in specific seasons may help forensic entomologists infer potential post-mortem movements of the remains, as well as estimate the mPMI or TOC. If the environmental impact on colonization is better understood, it will be possible to refine the estimation of time of colonization and provide more robust support to the investigators.

## Figures and Tables

**Table 1:** Location, level designation, elevation, and geographical coordinates of collection sites.

Location	Level	Elevation (m)	Coordinates
***Sicily***			
Catania	1	20	37.5427° N, 15.0843° E
San Teodoro	2	700	37.8071° N, 14.6994° E
Contrada Casale Nuovo	3	1153	37.8521° N, 14.6919° E
Monte Soro	4	1552	37.9258° N, 14.6702° E
***Ecuador***			
Tena	1	561	0.5938° S, 77.4818° W
Sarayacu	2	1312	0.4208° S, 77.4844° W
Baeza	3	1948	0.2759° S, 77.5321° W
Papallacta	4	3336	0.2225° S, 78.0825° W

**Table 2:** Average temperature (±SE), average relative humidity (±SE), and annual precipitation.

Location	Elevation Level	Elevation(m)	Temperature (°C) (±SE)	Humidity (%) (±SE)	Precipitation
***Sicily***					
Catania	1	20	25.23 ± 0.64	64.13 ±1.24	0.3 mm
San Teodoro	2	700	22.46 ± 0.83	62.06 ± 1.11	7 mm
Contrada Casale Nuovo	3	1153	19.72 ± 0.42	61.14 ± 0.87	8 mm
Monte Soro	4	1552	16.32 ± 0.13	62.2 ± 1.54	8.2 mm
***Ecuador***					
Tena	1	561	23.28 ± 1.2	99.52 ± 0.44	4330 mm
Sarayacu	2	1312	19.47 ± 0.66	94.57 ± 2.43	1013 mm
Baeza	3	1948	17.07 ± 0.87	89.37 ± 1.56	2220 mm
Papallacta	4	3336	11.29 ± 0.11	93.41 ± 1.32	1281 mm

**Table 3:** Relative abundance (%) of blow fly species collected in Sicily. Relative abundances are shown by species at each elevation as well as overall relative abundance (Rel. Ab.) during the duration of the survey.

SICILY						
Species	Elevation 20 m	Elevation 700 m	Elevation 1153 m	Elevation 1552 m	Total	Rel. Ab.
*Lucilia sericata*	42.23%	89.80%	43.22%	-	3375	68.50%
*Calliphora vicina*	56.79%	5.44%	50.60%	40.80%	1030	21.00%
*Calliphora vomitoria*	-	<1%	2.60%	55.16%	251	5.09%
*Lucilia ampullacea*	-	2.83%	2.78%	<1%	121	2.40%
*Lucilia silvarum*	<1%	2.09%	<1%	-	67	1.36%
*Chrysomya albiceps*	-	<1%	<1%	-	27	0.55%
*Calliphora subalpina*	-	<1%	-	3.78%	19	0.38%
*Lucilia caesar*	-	<1%	<1%	-	14	0.28%
*Lucilia illustris*	-	<1%	-	-	12	0.24%
*Lucilia cuprina*	<1%	<1%	<1%	-	7	0.14%
*Calliphora loewi*	-	<1%	<1%	-	2	0.04%
*Cynomia mortuorum*	-	<1%	-	-	1	0.02%
Number of Species	4	12	9	4		
Number of Specimens	206	3108	1150	397	4244	100.00%

**Table 4:** The male-to-female (m/f) ratio of adult blow flies found in Sicily. Ratios are depicted by species for each elevation, as well as an overall m/f ratio.

Species	Elevation 20 m	Elevation 700 m	Elevation 1153 m	Elevation 1552 m	Total Males	Tot Females	M/F Ratio
*Lucilia sericata*	13M/74F	147M/2644F	16M/481F		176	3199	0.05
*Calliphora vicina*	44M/73F	72M/97F	195M/387F	49M/113F	360	670	0.54
*Calliphora vomitoria*		2F	13M/17F	55M/164F	68	183	0.37
*Lucilia ampullacea*		38M/50F	7M/25F	1F	45	75	0.6
*Lucilia silvarum*	1F	14M/51F	1F		14	53	0.26
*Chrysomya albiceps*		26F	1F		0	27	
*Calliphora subalpina*		4F		6M/9F	6	13	0.46
*Lucilia caesar*		3M/7F	4M		7	7	1
*Lucilia illustris*		4M/8F			4	8	0.5
*Lucilia cuprina*	1M	4F	2F		1	6	0.16
*Calliphora loewi*		1F	1F		0	2	
*Cynomia mortuorum*		1F			0	1	

**Table 5:** Differences in species abundance across elevations in Sicily using Kruskal–Wallis one-way ANOVA and Dunn test with Benjamini–Hochberg adjustment.

Species	Kruskal–Wallis	Dunn					
		**1 v 2**	**1 v 3**	**1 v 4**	**2 v 3**	**2 v 4**	**3 v 4**
*L. silvarum*	6.771 (0.080)	-	-	-	-	-	-
***L. sericata***	**13.224 (0.004)**	**0.014**	0.211	0.670	0.210	**0.007**	0.114
*L. ampullacea*	7.313 (0.063)	-	-	-	-	-	-
*L. cuprina*	1.187 (0.756)	-	-	-	-	-	-
*L. illustris*	6.400 (0.094)	-	-	-	-	-	-
*L. caesar*	3.845 (0.279)	-	-	-	-	-	-
*C. vicina*	7.318 (0.062)	-	-	-	-	-	-
*C. vomitoria*	2.522 (0.471)	-	-	-	-	-	-
*C. subalpina*	6.450 (0.090)	-	-	-	-	-	-
*C. loewi*	2.143 (0.543)	-	-	-	-	-	-
*C. albiceps*	4.393 (0.222)	-	-	-	-	-	-
*C. mortuorum*	4.393 (0.222)	-	-	-	-	-	-

Numbers 1–4 relate to elevation level (1 = 20 m, 2 = 700 m, 3 = 1152 m, 4 = 1553 m). For Kruskal–Wallis, *p* values are in parentheses; number listed under Dunn = *p* value. Bold values represent significant values.

**Table 6:** Indicator species for two of the four elevations in Sicily. A level designation, elevation, indicator value, and *p* value are provided for each species.

Species	Level	Elevation	Value	*p* Value
*Lucilia sericata*	2	700 m	82.70	0.004
*Lucilia silvarum*	2	700 m	72.76	0.033
*Calliphora vicina*	3	1153 m	56.50	0.026

**Table 7:** Significant differences in blow fly communities in Sicily across elevations using the Multiple Response Permutation Procedure.

	Elevation 20 m	Elevation 700 m	Elevation 1152 m
**Elevation 700 m**	A: 0.1982; Obs. Delta: 0.5097; Exp. Delta: 0.6357 *p* = 0.13		
**Elevation 1152 m**	A: 0.0042; Obs. Delta: 0.5053; Exp. Delta: 0.5075 *p* = 0.6	A: 0.3035; Obs. Delta: 0.3681; Exp. Delta: 0.5285 ***p* = 0.035**	
**Elevation 1553 m**	A: −0.002; Obs. Delta: 0.7864; Exp. Delta: 0.7848 *p* = 0.66	A: 0.3732; Obs. Delta: 0.2714; Exp. Delta: 0. 5925 *p* = 0.2	A: 0.1968; Obs. Delta: 0.3648; Exp. Delta: 0. 4541 *p* = 0.2

**Table 8:** Relative abundance (%) of blow fly species collected in Ecuador. Relative abundances are shown by species at each elevation, as well as overall relative abundance (Rel. Ab.) throughout the duration of the survey.

Species	Elevation 561 m	Elevation 1312 m	Elevation 1948 m	Elevation 3336 m	Total	Rel. Ab.
*Consomyiops verena*	-	-	65.27%	74.90%	771	51.67%
*Calliphora nigribasis*	-	-	-	24.18%	138	9.25%
*Lucilia eximia*	25.12%	27.69%	2.72%	-	112	7.50%
*Chrysomya albiceps*	20.46%	43.85%	<1%	<1%	108	7.24%
*Paralucilia* sp.	31.63%	15.38%	-	-	88	5.90%
*Lucilia purpurescens*	-	2.30%	13.82%	-	79	5.30%
*Hemilucilia semidiaphana*	12.09%	3.08%	5.09%	-	58	3.90%
*Chrysomya megacephala*	3.72%	-	7.81%	-	51	3.42%
*Hemilucilia segmentaria*	2.79%	<1%	<1%	-	43	2.90%
*Lucilia ibis*	-	1.54%	4.00%	-	24	1.60%
*Chloroprocta idiodea*	-	4.61%	-	-	6	0.40%
*Lucilia* sp.	1.86%	-	<1%	-	5	0.33%
*Cochliomyia macellaria*	1.4%	<1%	-	-	4	0.27%
*Lucilia albofusca*	<1%	<1%	-	-	2	0.14%
*Cochliomyia hominivorax*	<1%	-	-	-	1	0.06%
*Chrysomya* sp.	-	-	<1%	-	1	0.06%
*Lucilia ochicornis*	-	-	<1%	-	1	0.06%
Number of Species	10	10	11	3		
Number of Specimens	215	130	550	550	1492	100.00%

**Table 9:** The male-to-female (m/f) ratio of adult blow flies found in Sicily. Ratios are depicted by species for each elevation, as well as an overall m/f ratio.

Species	Elevation 561 m	Elevation 1312 m	Elevation 1948 m	Elevation 3336 m	Tot Male	Tot Female	M/F Ratio
*Consomyiops verena*			72M/287F	91M/321F	163	608	0.27
*Calliphora nigribasis*				53M/80F	53	80	0.66
*Lucilia eximia*	8M/46F	3M/32F	15F		19	93	0.12
*Chrysomya albiceps*	8M/36F	13M/44F	2F	5F	21	87	0.24
*Paralucilia* sp.	13M/55F	20F			13	75	0.17
*Lucilia purpurescens*		3F	7M/69F		7	72	0.09
*Hemilucilia semidiaphana*	4M/22F	4F	5M/23F		9	49	0.15
*Chrysomya megacephala*	1M/7F		6M/37F		7	44	0.16
*Hemilucilia segmentaria*	1M/5F	1F	2F		9	34	0.12
*Lucilia Ibis*		2F	3M/19F		3	21	0.14
*Chloroprocta idiodea*		6F			0	6	
*Lucilia* sp.	4F		1F		0	5	
*Cochliomyia macellaria*	3F	1F			0	4	
*Lucilia albofusca*	1F	1F			0	2	
*Cochliomyia hominivorax*	1F				0	1	
*Chrysomya* sp.			1F		0	1	
*Lucilia ochicornis*			1F		0	1	

**Table 10:** Differences in species abundance across elevations using Kruskal–Wallis one-way ANOVA and Dunn test with Benjamini–Hochberg adjustment.

Species	Kruskal–Wallis	Dunn					
		1 v 2	1 v 3	1 v 4	2 v 3	2 v 4	3 v 4
***C. nigribasis***	**10.253** **(0.020)**	1.0	1.0	**0.017**	1.0	**0.026**	0.054
*C. megacephala*	5.025 (0.170)	-	-	-	-	-	-
*C. idiodea*	3.00 (0.392)	-	-	-	-	-	-
***C. albiceps***	**11.061 (0.011)**	0.848	**0.038**	**0.047**	0.067	0.057	1.0
*Chrysomya* sp.	3.00 (0.392)	-	-	-	-	-	-
*C. hominivorax*	3.00 (0.392)	-	-	-	-	-	-
*C. macellaria*	4.393 (0.222)	-	-	-	-	-	-
***C. verena***	**10.923 (0.010)**	1.0	0.074	**0.026**	0.099	0.053	0.615
*H. segmentaria*	2.987 (0.394)	-	-	-	-	-	-
***H. semidiaphana***	**8.754 (0.030)**	0.124	0.937	0.115	0.111	0.760	0.071
*L. albofusca*	2.143 (0.543)	-	-	-	-	-	-
*L. eximia*	5.67 (0.129)	-	-	-	-	-	-
***L. ibis***	**8.137 (0.040)**	0.288	**0.044**	1.0	0.411	0.360	0.088
*L. ochicornis*	3.00 (0.392)	-	-	-	-	-	-
***L. purporsecens***	**12.913 (<0.001)**	0.706	**0.006**	1.0	**0.023**	0.882	**0.013**
*Lucilia* sp.	7.702 (0.053)	-	-	-	-	-	-
***Paralucilia* sp.**	**11.331 (0.010)**	0.441	**0.018**	**0.036**	0.098	0.130	1.0

Numbers 1–4 = elevation levels (1 = 561 m, 2 = 1312 m, 3 = 1948 m, 4 = 3336 m). Significant *p* values are bolded for each test.

**Table 11:** Indicator species for three of the four elevations in Ecuador. A level designation, elevation, indicator value, and *p* value are provided for each species.

Species	Level	Elevation	Value	*p* Value
*Paralucilia* sp.	1	560 m	77.27	0.021
*Lucilia purpurescens*	3	1948 m	96.20	0.003
*Lucilia ibis*	3	1948 m	68.75	0.028
*Calliphora nigribasis*	4	3336 m	75.00	0.033

**Table 12:** Significant differences in blow fly communities in Ecuador across elevations using the Multiple Response Permutation Procedure.

	Elevation 561 m	Elevation 1312 m	Elevation 1948 m
**Elevation 1312 m**	A: 0.0108; Obs. Delta: 0.5555; Exp. Delta: 0.5616 *p* = 0.39		
**Elevation 1948 m**	A: 0.2893; Obs. Delta: 0.5465; Exp. Delta: 0.7689 ***p* = 0.027**	A: 0.3161; Obs. Delta: 0.5185; Exp. Delta: 0.7581 ***p* = 0.025**	
**Elevation 3336 m**	A: −0.2747; Obs. Delta: 0.586; Exp. Delta: 0.808 ***p* = 0.04**	A: 0.2977; Obs. Delta: 0.558; Exp. Delta: 0. 7945 ***p* = 0.028**	A: 0.1031; Obs. Delta: 0.5489; Exp. Delta: 0.612 *p* = 0.083

## Data Availability

The original data presented in the study are openly available in Zenodo at https://doi.org/10.5281/zenodo.7062111 and https://doi.org/10.5281/zenodo.7060325.

## Supplementary Materials

The following supporting information can be downloaded at: https://www.mdpi.com/article/10.3390/insects16050498/s1, Table S1: Sicily_Coverage_Based R/E; Table S2: Sicily_Size_Based_R/E; Table S3: Ecuador_Coverage_Based R/E; Table S4: Ecuador_Size_Based R/E; Figure S1: Sicily_Sample Completeness Curve; Figure S2: Sicily_Coverage_Based R/E_Sampling_Curve; Figure S3: Ecuador_Sample_Completeness_Curve; Figure S4: Ecuador_Coverage_Based_R/E Curve.
