# Correction: Gemmellaro et al. Species Richness and Distribution of Calliphoridae Along an Elevation Gradient in Sicily (Italy) and Ecuador. *Insects* 2025, *16*, 498

**DOI:** 10.3390/insects16070681

**Published:** 2025-06-30

**Authors:** M. Denise Gemmellaro, Gail S. Anderson, George C. Hamilton, Mariela Domínguez-Trujillo, Lauren M. Weidner

**Affiliations:** 1 Kean University, 1000 Morris Ave, Union, NJ 07083, USA; 2 Simon Fraser University, 8888 University Dr W, Burnaby, BC V5A 1S6, Canada; gail_anderson@sfu.ca; 3 Rutgers, The State University of New Jersey, 96 Lipman Drive, New Brunswick, NJ 08901, USA; hamilton@njaes.rutgers.edu; 4 Laboratorio de Entomología, Museo de Zoología QCAZ, Pontificia Universidad Católica del Ecuador, Quito 170143, Ecuador; mariela.adt@gmail.com; 5 Laboratorio de Zoología Terrestre, Instituto de Biodiversidad Tropical IBIOTROP, Colegio de Ciencias Biológicas y Ambientales, Universidad San Francisco de Quito USFQ, Quito 170901, Ecuador; 6 Arizona State University, West Campus, 4701 W Thunderbird Road, Glendale, AZ 85306, USA; lauren.weidner@asu.edu

## Missing Funding

In the original publication [1], the funder, The Pontificia Universidad Católica del Ecuador, which provided funds for the Forensic Entomology Project used to carry out the monitoring in Ecuador (PUCE: N13456A), was not included.

## Missing Acknowledgements

In the original publication [1], acknowledgements were not included. We wanted to thank the Entomology Laboratory at the Pontificia Universidad Católica del Ecuador (PUCE), especially Álvaro Barragán, who led and directed the Forensic Entomology Project. We also wanted to thank Saúl Aguirre, Ana Torres-Donoso and Emilia Moreno for their collaboration in the fieldwork and identification of samples in the Ecuador study. 

## Email Replacement

Replacing the email address of author Lauren M. Weidner:

lauren.weidner@asu.com

with

lauren.weidner@asu.edu

The authors state that the scientific conclusions are unaffected. This correction was approved by the Academic Editor. The original publication has also been updated.
