# Supplementary figures and images for: Species Richness and Distribution of Calliphoridae Along an Elevation Gradient in Sicily (Italy) and Ecuador
Source: Insects. 2025 May 6;16(5):498. doi: 10.3390/insects16050498 (PMC12112086; doi:10.3390/insects16050498)

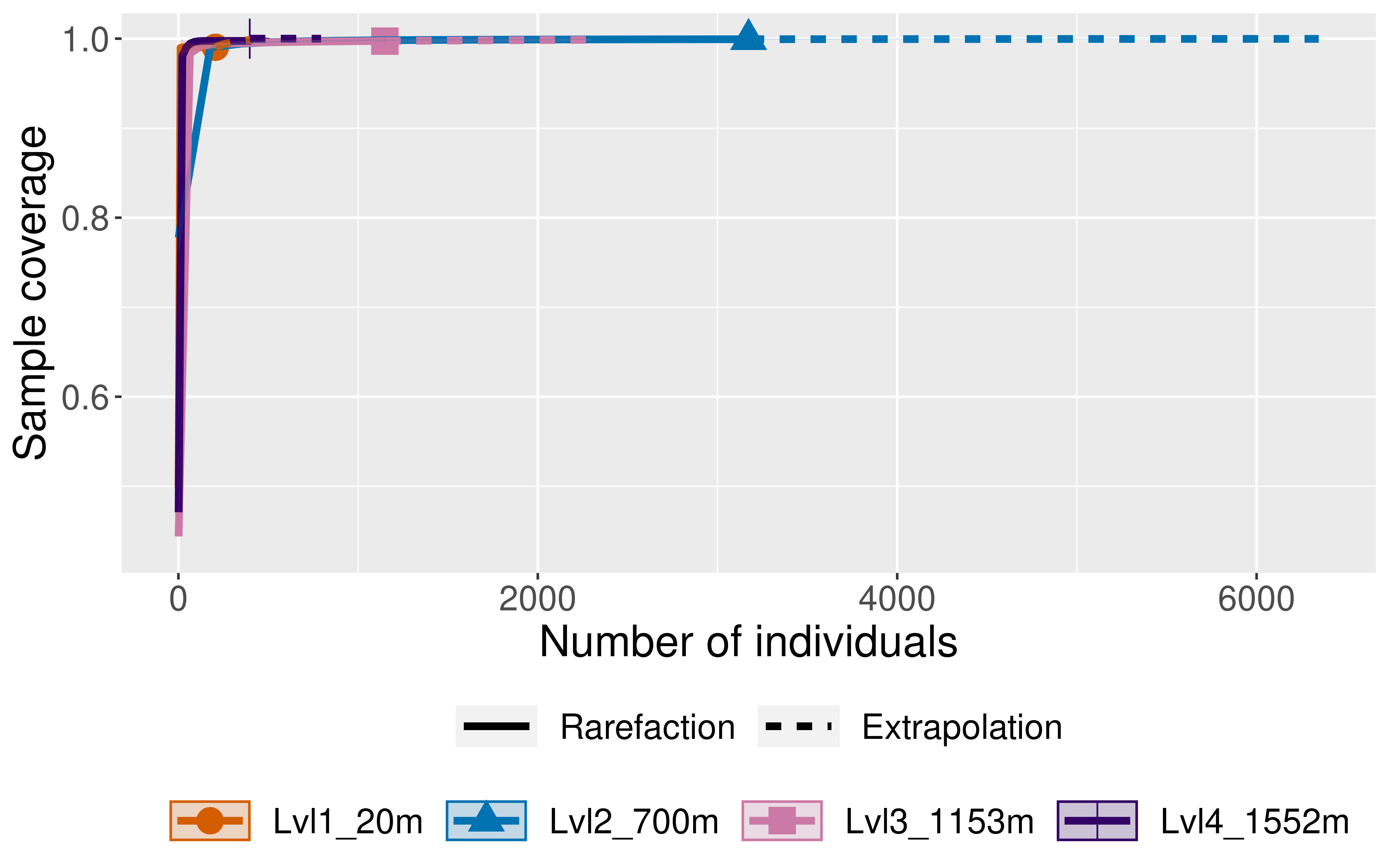

Supplement: Supplementary file 1 [file insects-16-00498-s001.zip › Fig_S1_Sicily_Sample Completeness._Curve.png]

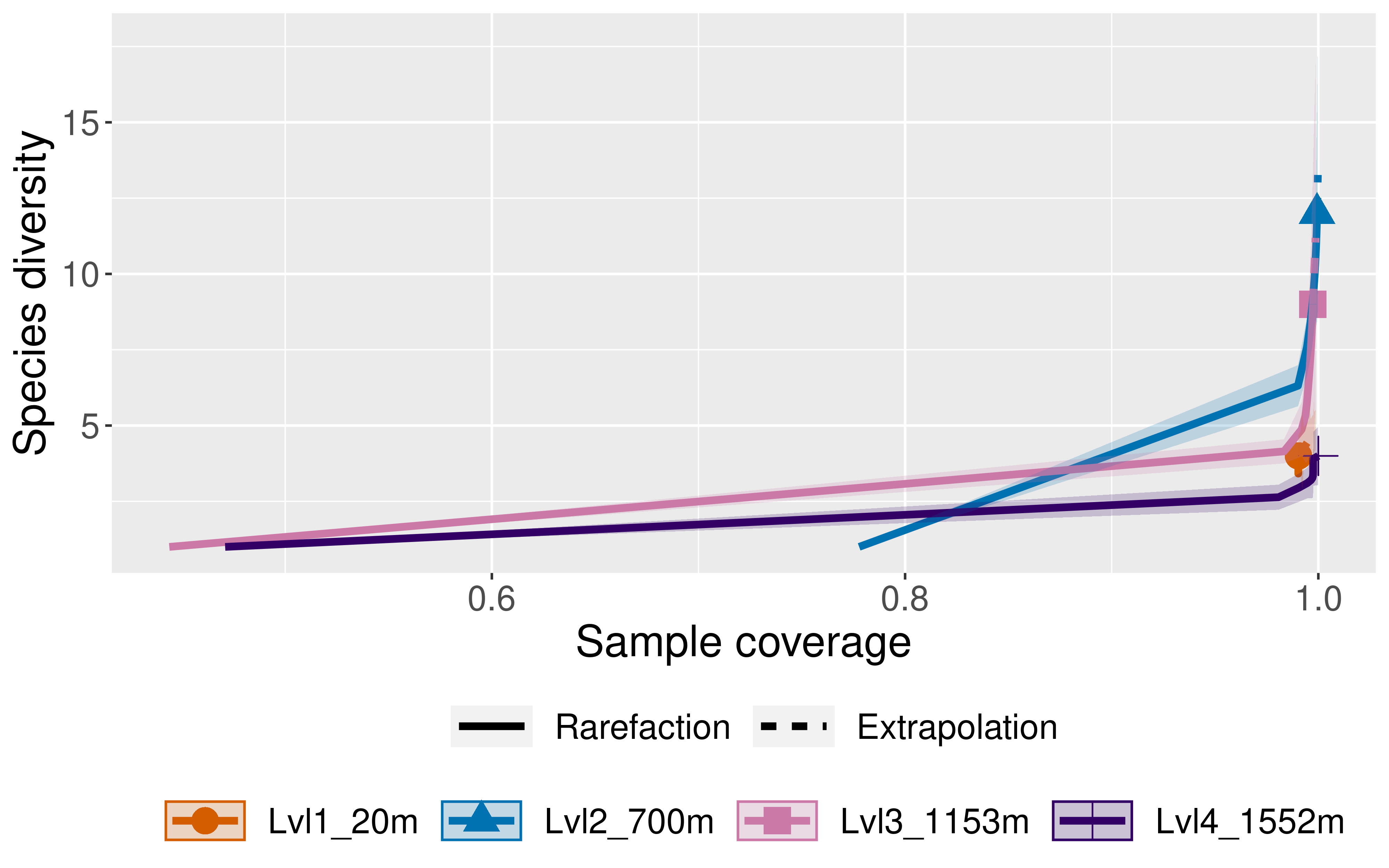

Supplement: Supplementary file 1 [file insects-16-00498-s001.zip › Fig_S2_Sicily_Coverage_Based R_E. Curve.png]

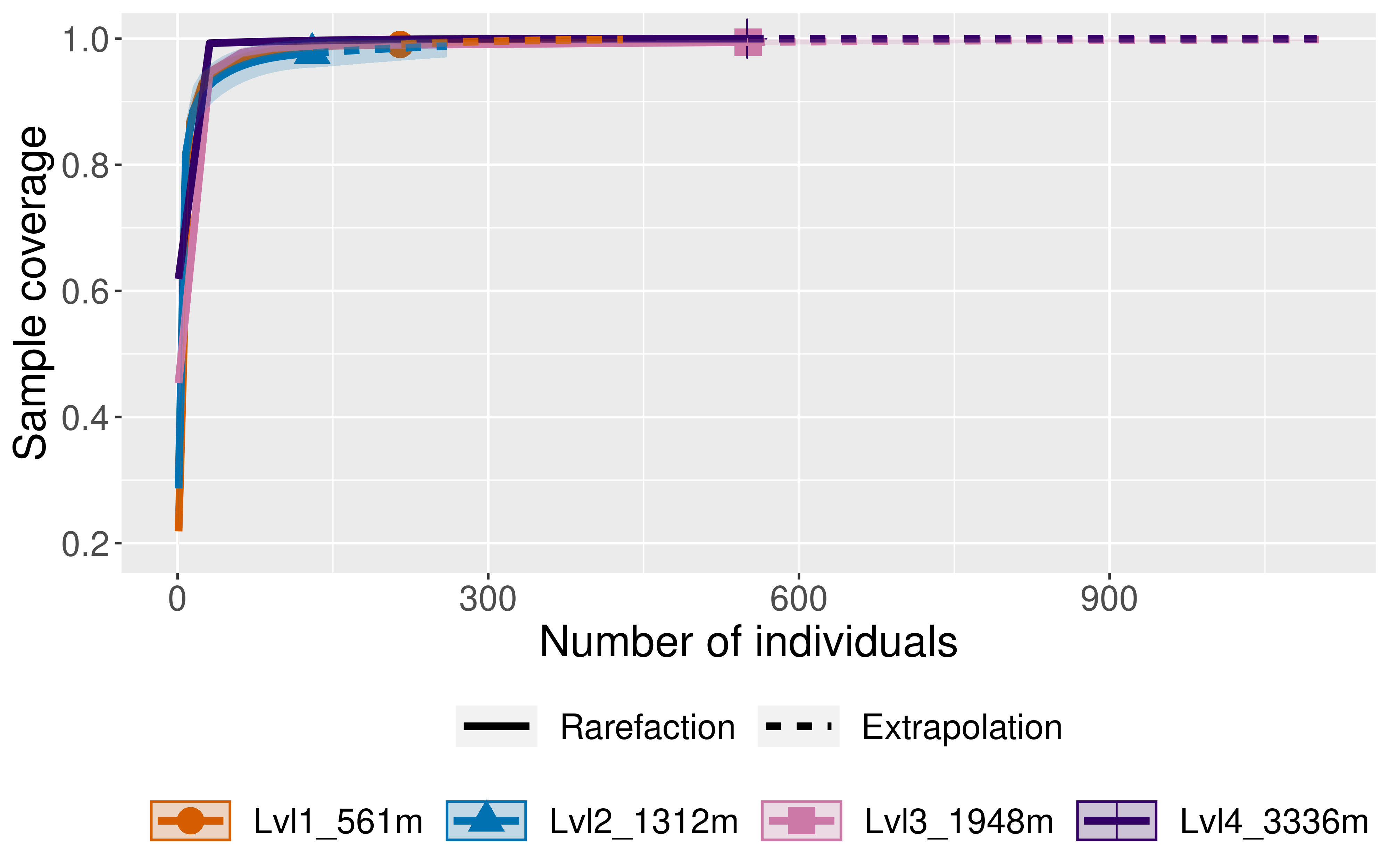

Supplement: Supplementary file 1 [file insects-16-00498-s001.zip › Fig_S3_Ecuador_Sample_Completeness_Curve.png]

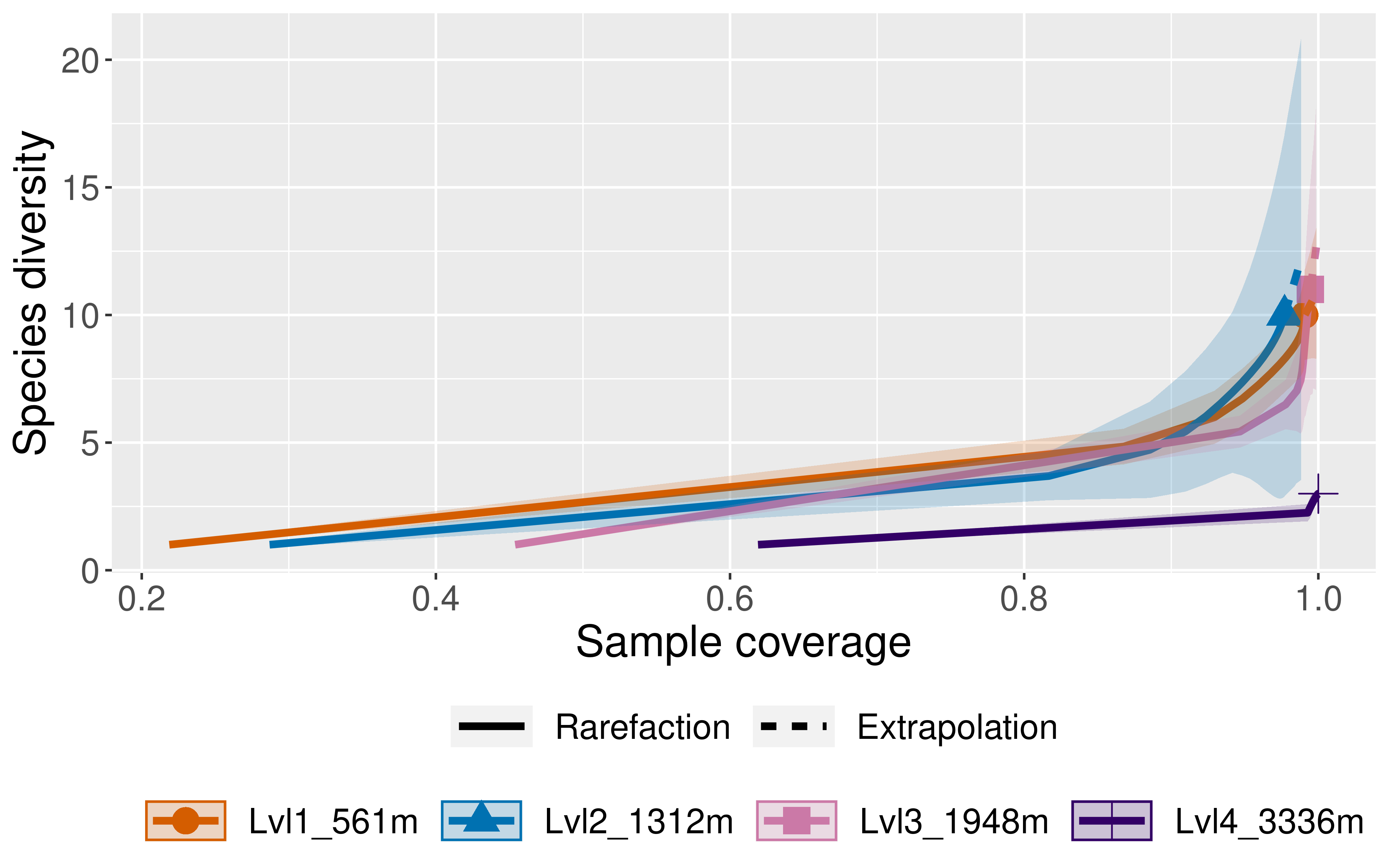

Supplement: Supplementary file 1 [file insects-16-00498-s001.zip › Fig_S4_Ecuador_Coverage_Based_R_E_Curve.png]
